# Supplementary material for: Synaptic cell adhesion molecule Cdh6 identifies a class of sensory neurons with novel functions in colonic motility
Source: bioRxiv. 2024 Aug 8:2024.08.06.606748. Preprint. [Version 1] doi: 10.1101/2024.08.06.606748 (PMC11326146; doi:10.1101/2024.08.06.606748)
Supplement: Supplement 1 [file NIHPP2024.08.06.606748v1-supplement-1.pdf]

bioRxiv preprint doi: https://doi.org/10.1101/2024.08.06.606748; this version posted August 8, 2024. The copyright holder for this preprint (which was not certified by peer review) is the author/funder, who has granted bioRxiv a license to display the preprint in perpetuity. It is made available under aCC-BY 4.0 International license.

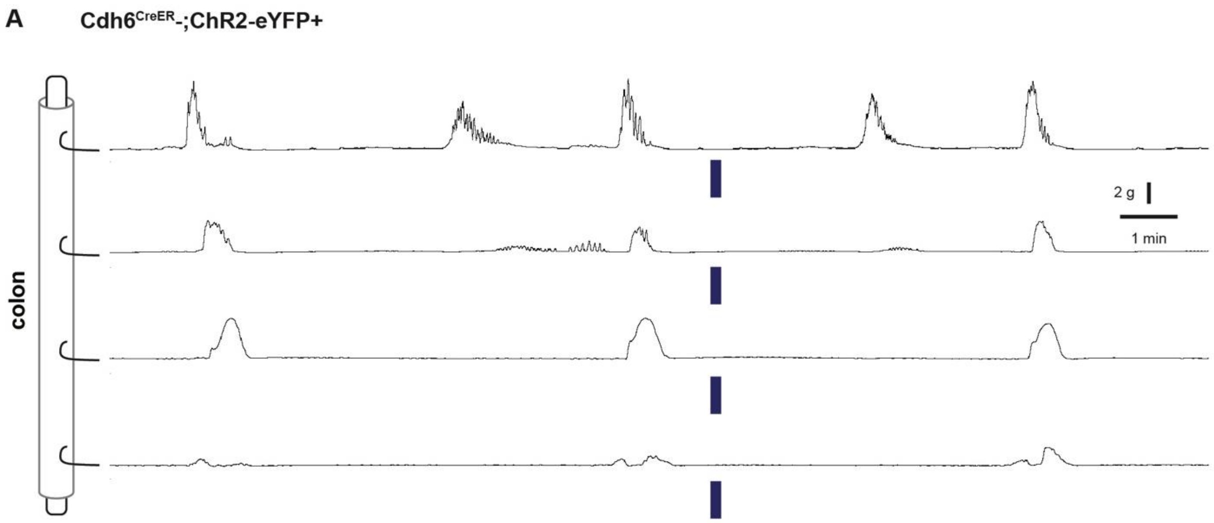

Fig. S1.

Optogenetic stimulation control. (**A**) Representative force traces of control Cdh6^{CreER}-;Chr2eYFP+ colon (n = 5). Blue bars indicate timing of light stimulation. LEDs placed distal to distal hook.

bioRxiv preprint doi: https://doi.org/10.1101/2024.08.06.606748; this version posted August 8, 2024. The copyright holder for this preprint (which was not certified by peer review) is the author/funder, who has granted bioRxiv a license to display the preprint in perpetuity. It is made available under aCC-BY 4.0 International license.

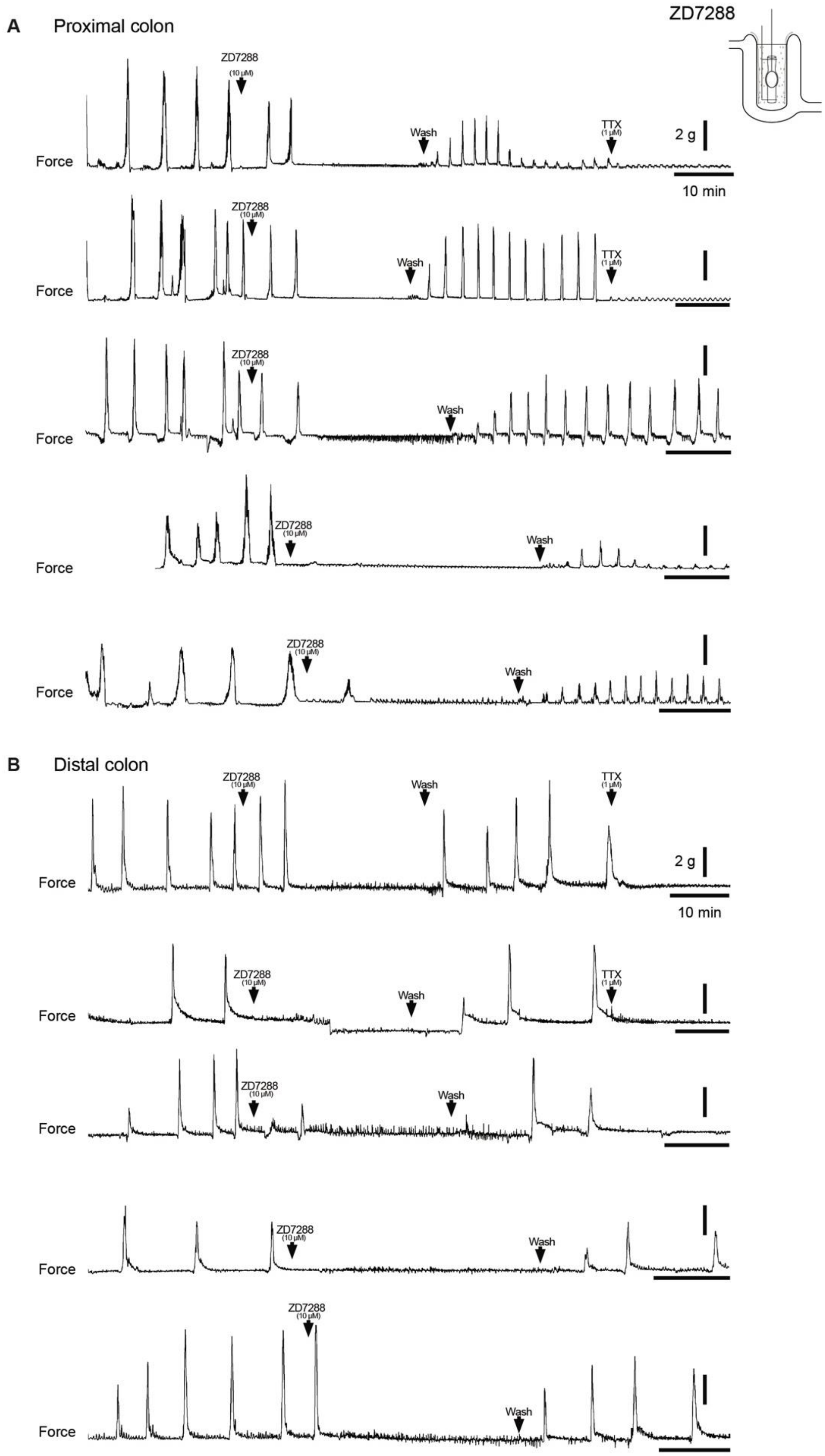

725

bioRxiv preprint doi: https://doi.org/10.1101/2024.08.06.606748; this version posted August 8, 2024. The copyright holder for this preprint (which was not certified by peer review) is the author/funder, who has granted bioRxiv a license to display the preprint in perpetuity. It is made available under aCC-BY 4.0 International license.

Fig. S2. Pharmacologic blockade of I_H with ZD7288 abolishes spontaneous CMCs. (**A, B**) Representative force traces from tethered pellet in proximal half (A) or distal half (B) of colon. Addition of 10 µM ZD7288 (first arrowhead), followed by washout in Krebs (second arrowhead), and addition of 1 µM TTX (third arrowhead). Scale bars represent 2 g force (vertical bars) and 10 minutes (horizontal bars) for all traces.

bioRxiv preprint doi: https://doi.org/10.1101/2024.08.06.606748; this version posted August 8, 2024. The copyright holder for this preprint (which was not certified by peer review) is the author/funder, who has granted bioRxiv a license to display the preprint in perpetuity. It is made available under aCC-BY 4.0 International license.

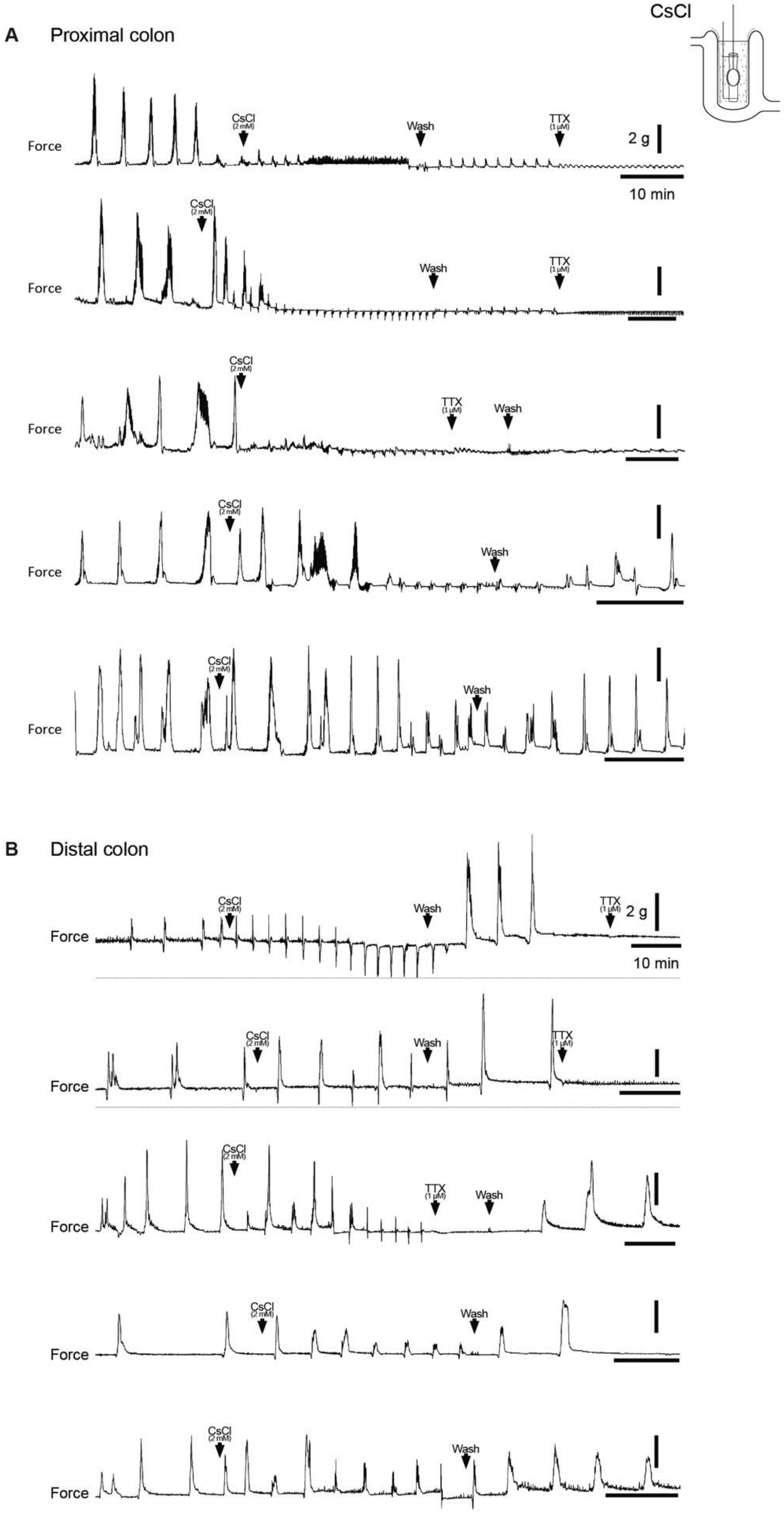

733

bioRxiv preprint doi: https://doi.org/10.1101/2024.08.06.606748; this version posted August 8, 2024. The copyright holder for this preprint (which was not certified by peer review) is the author/funder, who has granted bioRxiv a license to display the preprint in perpetuity. It is made available under aCC-BY 4.0 International license.

Fig. S3. Pharmacologic blockade of I_H with CsCl impairs generation of CMCs. (A, B) Representative force traces from tethered pellet in proximal half (A) or distal half (B) of colon. Addition of 2 mM CsCl (first arrowhead), followed by washout in Krebs (second arrowhead), and addition of 1 µM TTX (third arrowhead). Scale bars represent 2 g force (vertical bars) and 10 minutes (horizontal bars) for all traces.
